# The Short Warwick‐Edinburgh Mental Wellbeing Scale in Swedish: A Large‐Scale Study of Reliability and Validity in Adults

**DOI:** 10.1002/hsr2.73089

**Published:** 2026-08-19

**Authors:** Frida Ekbom Lodin, Johnny Pellas

**Affiliations:** ^1^ Habilitation Services, Uppsala County Uppsala Sweden; ^2^ Department of Public Health and Caring Sciences Uppsala University Uppsala Sweden

**Keywords:** mental health, psychological well‐being, psychometrics, validation

## Abstract

**Background and Aims:**

The Short Warwick‐Edinburgh Mental Wellbeing Scale, SWEMWBS, is a self‐rating scale designed to measure positive aspects of mental health. The aim of the present study was to investigate the psychometric properties of the official Swedish translation of the SWEMWBS within a representative sample of Swedish adults.

**Methods:**

Data from 31,802 participants aged 18–102 years were retrieved from the public health survey Life and health. Reliability was investigated by examining the internal consistency with McDonald's omega in different age groups. Convergent validity was evaluated by examining Spearman's rho correlations with the six‐item Kessler psychological distress scale (Kessler‐6). Structural validity was investigated through exploratory and confirmatory factor analysis, and measurement invariance across age groups was examined using multi‐group confirmatory factor analysis.

**Results:**

The internal consistency was strong, with omega values ranging from 0.87 to 0.89 in the different age groups. The correlations between SWEMWBS and Kessler‐6 in the different age groups were statistically significant and strong, ranging from −0.62 to −0.75. The factor analyses offered support to the single‐dimension structure but showed better fit for a two‐factor model. Measurement invariance testing demonstrated stable psychometric properties across age groups.

**Conclusions:**

The official Swedish translation of SWEMWBS has strong reliability and convergent validity. Although the two‐factor model showed superior fit, a strong correlation between factors indicated substantial overlap suggesting that the scale could be considered unidimensional, in line with previous research. The difference in factor structure compared to the original English version might be due to language differences or the gathering of data during the covid‐pandemic. The most noticeable strength of the current study is the large age‐stratified sample, including the oldest old.

## 1 Introduction

Mental wellbeing is the positive aspect of mental health. The World Health Organization (WHO) has stated that positive mental health is the “foundation for wellbeing and effective functioning for both the individual and the community” and says it is a state “which allows individuals to realize their abilities, cope with the normal stressors of life, work productively and fruitfully and make a contribution to their community” [1]. What is stated in the last couple of sentences is what clinicians and policy makers want for the people they serve, not “just” the absence of mental illness.

Mental health fluctuates from time to time since it depends on both our internal and external circumstances, which change [2]. Our mental health also influences how well we cope with challenges which in turn influences the chances of feeling well in the future. Mental health influences for example physical health and relationships. Programs designed to promote mental health aim to increase the number of people who feel mentally well and to increase the amount of time they feel like that. Mental wellbeing is a construct that includes both the subjective experience of positive affect (the hedonic perspective) and psychological functioning and self‐realization (the eudaimonic perspective) [3].

There are multiple validated measures of mental illness playing an important role in as well research as in clinical practice involving individuals and groups. They are often a part of the diagnostic procedure as well as in the evaluation of interventions. In clinical practice, in research and in monitoring mental health at population level one has a lot to gain from also using a measure of mental wellbeing. Some have even argued that we should consider positive mental health as a separate continuum to mental illness, e.g. “The complete state model of mental health” [4]. Others view mental health as a single continuum, with mental illness at one end, and mean that people with mental illness can experience wellbeing when their illness is not making them feel bad or function poorly [2]. There are advantages to including a measure of wellbeing in both research and clinical practice. These include the fact that mental wellbeing is more than the absence of mental illness. A measure of wellbeing can help detect suffering that is subclinical, which diagnostic measures are not construed to find, but is important to detect in order to prevent a worsening in mental health. Another advantage is that regardless of whether one considers positive mental health as a continuum, separate to one of mental illness, or see it as the opposite to mental illness, it is a fact that people can experience some degree of mental illness and a degree of positive mental health simultaneously (i.e., there is an overlap) and it can help drive positive action and change if one also puts an emphasis on positive mental health. That is, there is a risk of missing important information if one only emphasizes on symptoms of mental illness. In for example treatment in line with Acceptance and Commitment Therapy, ACT, it would also undoubtedly be contradictory to only evaluate treatment success by measuring the degree of symptoms since the rationale behind this treatment approach stresses that success means living well *with* feelings of for example anxiety rather than getting rid of those feelings [5]. There are a couple of measures of self‐rated wellbeing that have been developed in Sweden or translated into Swedish. Most of these focus on emotional wellbeing (the hedonic perspective) and others include psychological functioning (the eudaimonic perspective). Not all measures translated into Swedish have been validated to make sure they capture what the measures are supposed to capture.

One instrument that has been developed to capture wellbeing and include both the hedonic and eudaimonic perspective is The Warwick‐Edinburgh Mental Wellbeing Scale (WEMWBS). The scale has been extensively validated in different cultures and settings, has been translated into 31 languages and is easy to complete and score [2]. WEMWBS was developed in the UK in 2007 by a group of experts from adjacent disciplines based on current academic literature, qualitative work with focus groups and psychometric evaluation of an existing scale that was similar to what they wanted but failed in some important aspects [6]. The scale consists of 14 items all positively worded and using a 5‐point Likert scale for response alternatives. The original validation showed a good content validity, good face validity, good criterion validity, good internal consistency and reliability and support for the hypothesized single‐factor model [6]. It did not show a ceiling effect in a general population sample and was less affected by social desirability than is the case with many similar scales. WEMWBS covers multiple aspects of psychological functioning (the eudaimonic perspective): optimism, autonomy, agency, curiosity, clarity of thought and positive relationships and positive affect (the hedonic perspective): confidence, feeling relaxed, cheerful, having the energy to spare. The original developing team behind the scale noticed a risk of item redundancy. After a Rasch analysis in a later study, that intended to assess the internal construct validity of the 14‐item WEMWBS, a shorter version was developed, the 7‐item Short Warwick Edinburgh Mental Wellbeing Scale (SWEMWBS) [7]. The new shorter scale showed a correlation of 0.954 to the original version. It can be argued that the face validity of this shorter version is lower since it covers markedly more psychological functioning than positive affect, but it has better psychometric qualities. It has a good fit with the Rasch model and is strictly unidimensional, so it provides an interval scale estimate of wellbeing and thus is suited for parametric analysis according to the developers [7]. To use the parametric version, the raw total score must be transformed into a metric score using the conversion table provided in the original study. The metric scores were derived through a linear logit transformation of the raw scores. According to the developing team the correlation between wellbeing and mental illness is found to be high (0.6–0.9). They have established cut off‐points that can be used if it is important to focus on likely mental illness but it is used as a continuous measure of wellbeing in its own right [2].

The first attempt to validate SWEMWBS in Sweden was done by a group that measured the mental wellbeing of hotel managers [8]. The scale showed an adequate internal consistency and reliability. The confirmatory factor analysis showed moderate fit to the unidimensional model. The construct validity, criterion‐related validity and discriminant validity were acceptable. However, the study used a translation previous to the now official one. The official version was validated through a Rasch analyses that found an acceptable fit to the Rasch measurement model but also found a ceiling effect and concluded that there were large measurement uncertainties for most of the population [9]. A study on Swedish 15‐years olds investigated the effect of “straight lining” as a response pattern on the dimensionality and internal consistency of SWEMWBS [10]. Their results supported the unidimensional structure and showed good internal consistency. A study in the south of Sweden examined the psychometric properties of the Swedish translation in a sample of 5000 adolescents [11]. They combined classical test theory and Rasch analysis. Their results showed excellent internal consistency, acceptable construct validity and measurement invariance and confirmed the unidimensional structure.

The aim of the current study is to investigate the psychometric properties of the official Swedish translation of the SWEMWBS within a representative sample of Swedish adult citizens. This includes assessing reliability by examining internal consistency in different age groups, evaluating the convergent validity by examining the correlation with the six‐item Kessler psychological distress scale, and investigating the structural validity through factor analysis.

## 2 Methods

### 2.1 Participants and Procedure

Data for the present study were retrieved from the public health survey “Liv och Hälsa” (Eng. “Life and health”) which is conducted recurrently in the Swedish counties of Uppsala, Södermanland, Västmanland, Värmland and Örebro. Together these counties have approximately 1,2 million citizens. The survey was distributed to 78,482 randomly selected adult citizens in this region between February and June in 2022 and 33,342 answered the full version of the survey. Respondents were introduced to the survey via regular mail, but the survey was initially digital. Several measures were taken to ensure that everyone who wanted to fill in the survey could, for example the digital version was accessible in English and could also be used with a speech synthesis. In a later stage a version on paper was distributed (only in Swedish). The survey entailed a total of 68 questions plus sub‐questions and supplementary questions, and apart for the measures used for this study the survey comprised questions about living habits, impairments, living conditions (e.g., social and financial), work conditions, received care, health status and perceived health. The complete survey is described in detail elsewhere [12].

### 2.2 Measures

The Swedish version of the SWEMWBS used in “Liv och hälsa” and other large scale Swedish surveys is a translation conducted by the Public health agency of Sweden to be used in a survey in 2018. See Table 1 for how the questions in SWEMWBS are worded in the original English version. The scale is used in the article for reference purposes only, a license is required for further use of the scale. The respondent is asked to choose the response that describes their experience during the last 2 weeks. The response alternatives are: “all of the time”, “most of the time”, “some of the time”, “a little of the time” and “none of the time”. These are also the response alternatives of the Swedish version, but the order of the alternatives (as well as the scoring) is reversed in comparison to the original version in English. The scores in the Swedish version range from 7 (high wellbeing) to 35 (low wellbeing).

**Table 1:** Questions in the short warwick‐edinburgh mental wellbeing scale, SWEMWBS.

Item	
1.	I've been feeling optimistic about the future
2.	I've been feeling useful
3.	I've been feeling relaxed
4.	I've been dealing with problems well
5.	I've been thinking clearly
6.	I've been feeling close to other people
7.	I've been able to make up my own mind about things

The Kessler scales (the 6‐item version as well as the 10‐item version) were originally developed to be included in the US National Health Interview Survey, NHIS [13]. They were also later included in the WHOs World mental health Survey [14]. The scale is a global measure of psychological distress regarding complaints of depression and generalized anxiety that are common in, but not specific to any specific, psychiatric diagnosis. It was meant to play an important, but up till then missing, crosswalk between community and clinical epidemiology with a set of questions that define behavioral, emotional, cognitive and psychophysiological manifestations of psychological distress. Item selection was based om Item‐response theory to maximize measurement precision and Kessler‐6 has proved to be an efficient screening tool for serious mental illness [13]. The calibration study [13] showed that a cut point on 13+ on the Kessler‐6 is optimal for assessing the prevalence of serious mental illness in the national population. The Swedish version of Kessler‐6 that is used in “Liv och hälsa”, and other large scale Swedish surveys, is a translation by the Center for Epidemiology and Community Medicine [15]. Lundin et al [16] compared the Swedish translation of Kessler‐6 to the Swedish translation of the General Health Questionnaire‐12 to develop a score conversion table. The two scales were found to measure the same phenomenon. The six‐item scale used in this study consists of six questions asking about how much of the time during the last 30 days that the respondent has felt “nervous”, “hopeless”, “restless”, “so depressed that nothing could cheer them up”, that “everything was an effort” and “worthless”. The response alternatives are: “all of the time”, “most of the time”, “some of the time”, “a little of the time” and “none of the time”. The scores range from 0 to 24, with higher scores indicating higher levels of psychological distress.

### 2.3 Statistical Analyses

Missing data was handled using listwise deletion, whereby only participants with complete responses on all SWEMWBS items were included in the main analyses. To assess potential nonresponse bias, a missing data indicator variable was created distinguishing participants with complete SWEMWBS data from those with incomplete data. Differences between these groups were examined with respect to age, sex, and educational level. Age differences were analysed using the Mann–Whitney U test. Sex and educational level were assessed using chi‐square tests. Since the response alternatives in the Swedish version of the SWEMWBS are reversed compared to the original version, the items were reversed back before analysis to be able to make some comparisons to the English normative data. As recommended by the developers of SWEMWBS, the raw total scores were then transformed into metric scores using a conversion table, since this makes it possible to compare the results to previous studies [2]. Reliability of the SWEMWBS was calculated with McDonald's omega (ω), and the results were interpreted as follows: 0.65–0.80 acceptable, above 0.80 strong [17]. Correlations between SWEMWBS and Kessler‐6 were evaluated using Spearman's rho (r_s_) both for the total sample and for different age groups. When calculating the correlation with Kessler‐6 only participants that had a complete SWEMWBS and a complete Kessler‐6 were included. Correlation coefficients were interpreted as follows: 0.10 small, 0.30 medium, 0.50 large [18]. Structural validity was investigated using exploratory factor analysis (EFA) and confirmatory factor analysis (CFA). Kaiser‐Mayer‐Olkin‐analysis was performed to investigate sampling adequacy, and Bartlett's test of sphericity was used to investigate if items were sufficiently correlated for factor analyses. In the EFA, an initial unrotated analysis was performed to investigate possible number of factors, followed by an analysis with oblimin rotation with the number of factors from the initial analysis. Data was extracted using minimum residuals. Factors with an Eigenvalue of ≥ 1 consisting of at least two items with loadings ≥ 0.40 were used. The CFAs were conducted using the weighted least squares mean and variance adjusted estimator (WLSMV) based on polychoric correlations, which is appropriate for ordinal Likert‐type data. The CFA model fit was assessed with Chi square (*χ* ^2^), comparative fit index (CFI), the Tucker Lewis index (TLI), the root mean square error of approximation (RMSEA), and the standardized root mean squared error (SRMR). Scaled (i.e., robust) values for *χ* ^2^, CFI, TLI and RMSEA were reported. The fit indices were interpreted as follows: CFI and TLI, 0.96–0.99 = excellent, 0.90–0.95 acceptable; RMSEA and SRMR, 0.01–0.05 excellent, 0.06–0.08 acceptable [19]. Measurement invariance across age groups was examined using multi‐group CFA. A stepwise procedure was applied to test configural and metric invariance for both the one‐factor and two‐factor models. Model fit was evaluated using CFI, with changes in CFI (ΔCFI) of ≤ 0.01 used as the criterion for evaluating measurement invariance across models [20]. The statistical analyses were made using Jamovi version 2.7.33 (available from https://www.jamovi.org), with the SEM/lavaan module for the CFAs.

### 2.4 Ethics

The Life and health‐survey has been approved by the Swedish Ethical Review Authority (approval number 2021‐05814‐01). All participants consented to participate by returning the survey. All data from the survey is anonymized.

## 3 Results

Table 2 shows the demographic data for the sample. After listwise deletion, data from 31.802 participants (95.4%) was retained. Participants with incomplete SWEMWBS data were significantly older and had a lower educational level than those with complete data (*p* = < 0.001 for both tests). No differences were observed between groups with respect to sex (*p *= 0.51), indicating similar response patterns among men and women.

**Table 2:** Demographic statistics for the total sample and different age groups.

	*N*	Total sample	18–29	30–39	40–49	50–59	60–69	70–79	80–89	90+
Sex in %	31,802									
Female		54.1	60.8	60.1	58.8	54.6	53.7	49.9	46.7	49.8
Male		45.9	39.2	39.9	41.2	45.4	46.3	50.1	53.3	50.2
Age in years	31,802									
Mean		58.4	23.8	34.6	44.8	54.7	64.7	74.3	84.2	92.4
Standard deviation		19.9	3.7	2.9	2.9	2.8	2.9	2.8	2.8	2.5
Age distribution in %	31,802	100	12.4	8.8	11.1	14.1	16.8	23.3	11.2	2.2
Highest educational level in %	29,502									
Pre secondary		17.5	6.0	5.5	5.5	8.0	14.4	24.6	40.3	49.9
Upper secondary		44.4	43.5	37.0	39.9	51.2	50.7	45.8	36.9	30.8
Post secondary		38.0	50.5	57.5	54.6	40.8	34.9	29.5	22.9	19.3
Birth country in %	31,799									
Sweden		87.8	87.2	83.7	83.6	87.3	89.2	90.1	89.1	90.4
Other Nordic country		4	0.5	1.0	1.6	3.3	5.3	6.3	6.4	4.4
Other country		8.2	12.3	15.2	14.8	9.3	5.6	3.6	4.5	5.1

### 3.1 Descriptive Statistics and Internal Consistency

See Table 3 for means and standard deviations of SWEMWBS. The internal consistency was strong in the total sample as well as in the different age groups with values ranging from 0.87 to 0.89, see Table 3. The analysis showed that the removal of an item would not have made internal consistency higher.

**Table 3:** Means and standard deviations on SWEMWBS and Kessler‐6, and McDonald's omega internal consistency for the SWEMWBS, for the total sample and different age groups.

	SWEMWBS (*N* = 31,802)			Kessler‐6 (*N* = 30,861)
	Raw scores mean (SD)	Transformed scores mean (SD)	ω	Mean (SD)
Total	27.61 (4.41)	25.21 (4.28)	0.88	4.50 (4.46)
18–29	26.37 (5.06)	24.15 (4.64)	0.88	6.97 (5.16)
30–39	27.00 (4.73)	24.69 (4.51)	0.89	5.75 (4.66)
40–49	27.51 (4.51)	25.13 (4.34)	0.89	4.97 (4.48)
50–59	27.84 (4.26)	25.42 (4.21)	0.89	4.28 (4.27)
60–69	28.28 (3.99)	25.82 (4.07)	0.88	3.53 (3.82)
70–79	28.15 (3.91)	25.65 (3.96)	0.87	3.47 (3.86)
80–89	27.41 (4.44)	25.00 (4.26)	0.87	4.13 (4.35)
90+	26.22 (5.23)	24.02 (4.67)	0.89	4.76 (4,76)

Abbreviations: SWEMWBS = short warwick‐edinburgh mental wellbeing scale; SD = standard deviation; ω = McDonald's omega.

### 3.2 Convergent Validity

The correlations between SWEMWBS and Kessler‐6 in the sample were statistically significant and strong both for the total sample (r_s_ = −0.68) and for each age group (r_s_ between −0.62 and −0.75), see Table 4.

**Table 4:** Spearman's rho correlation coefficients between SWEMWBS and Kessler‐6 for the total sample and different age groups.

	*n*	Spearman's rho
Total	30,861	−0.68*
18–29	3,804	−0.75*
30–39	2,722	−0.73*
40–49	3,460	−0.70*
50–59	4,390	−0.69*
60–69	5,235	−0.65*
70–79	7,210	−0.64*
80–89	3,387	−0.64*
90+	653	−0.62*

* *p* < 0.01.

### 3.3 Factor Analyses

The sampling adequacy was acceptable (KMO = 0.9) and Bartlett's test of sphericity demonstrated that correlations between items were large enough for factor analysis (*χ* ^2^ [21] = 100897, *p* = < 0.001). The EFA showed that there were two factors that together explained 57% of the variance, with each factor explaining 35% and 22% respectively; factor one Eigenvalue = 2.44, factor two Eigenvalue = 1.56, see Table 5 for factor loadings. Based on previous research, a one‐factor model (model 1) was tested using CFA, as well as the two‐factor model (model 2) suggested by the EFA with factor one consisting of items 3, 4, 5 and 7, and factor 2 consisting of items 1, 2 and 6, factor loadings are displayed in Table 5. The one‐factor model showed mixed fit. SRMR indicated good fit (SRMR = 0.032), and both CFI and TLI indicated excellent fit (CFI = 0.993, TLI = 0.990). However, RMSEA indicated poor fit (RMSEA = 0.141). The scaled Chi‐square test was significant, *χ* ^2^ [14] = 8841, *p *< 0.001. The two‐factor model demonstrated improved fit. SRMR, CFI and TLI indicated excellent fit (SRMR = 0.020, CFI = 0.998, TLI = 0.996). RMSEA indicated unacceptable fit (RMSEA = 0.092). The scaled chi‐square test was significant, *χ* ^2^ [13] = 3496, *p *< 0.001. The latent factors were very highly correlated (r = 0.86, *p *< 0.001).

**Table 5:** Factor loadings for each item in the exploratory and confirmatory factor analyses.

	EFA		CFA		
			Model 1	Model 2	
Item	Factor 1	Factor 2	Factor 1	Factor 1	Factor 2
Item 1	0.17	0.61	0.77		0.82
Item 2	−0.05	0.83	0.73		0.77
Item 3	0.62	.016	0.80	0.81	
Item 4	0.75	0.08	0.87	0.89	
Item 5	0.89	−0.09	0.85	0.86	
Item 6	0.26	0.46	0.71		0.76
Item 7	0.61	0.08	0.73	0.75	

Abbreviations: EFA = exploratory factor analysis; CFA = confirmatory factor analysis.

### 3.4 Measurement Invariance

For the one‐factor model, configural invariance showed excellent fit across age groups (CFI = 0.97). Metric invariance resulted in CFI = 0.98, indicating no deterioration in fit (ΔCFI = 0.008), thereby supporting metric invariance. For the two‐factor model, configural invariance also demonstrated excellent fit (CFI = 0.99). Metric invariance remained stable (CFI = 0.99), with a negligible change in fit (ΔCFI = 0.003). The results support metric invariance across age groups for both factor solutions.

## 4 Discussion

The aim of the current study was to investigate the psychometric properties of the official Swedish translation of the SWEMWBS within a representative sample of Swedish adult citizens. This included assessing the reliability by examining internal consistency in different age groups, evaluating the convergent validity by examining the correlation with the six‐item Kessler psychological distress scale, and investigating the structural validity through exploratory and confirmatory factor analysis.

The official Swedish translation of SWEMWBS is used by the Public health agency of Sweden in regular large scale public health surveys since 2018. Since then, the translation has been validated in a couple of studies but this is by far the largest validation study of this translation to date. The mean and the standard deviation of SWEMWBS in the Swedish sample are similar to the UK norms that showed a mean of 23.5 and a standard deviation of 3.9 in the general population samples [2]. The results of this study show strong internal consistency and convergent validity, in line with previous research [8, 10, 11, 21]. The negative correlation with psychological distress was expected because comparisons of WEMWBS with scales of mental illness have, as mentioned, shown a high level of correlation in previous studies ranging from 0.6 to 0.9 [2]. The current study offered some support to the single‐dimension structure proposed by the original developing team and later confirmed by several other studies [7, 8, 10, 11], but showed better fit for a two‐factor model. However, the latent factors were very highly correlated (r = 0.86), indicating substantial overlap between the constructs and raising concerns regarding discriminant validity. This suggests that although the two‐factor model provides a better statistical representation of the data, the two dimensions may not represent distinct constructs. The optimized fit for the two‐factor model might reflect that the translation has affected the scale or that the factor structure is partially dependent on the culture in which the measure is used. There are, however, other studies that have also found more than one factor when using SWEMWBS, for example a recent Finnish study [21]. Item 3 in the current study loads on another factor compared to the Finnish study but there is a partial overlap in the findings between the current study and the Finnish. The two‐factor model proposed by the current study has a very similar degree of fit compared to the Finnish bifactor model. The mean values in the Swedish and Finnish samples are also quite similar. The comparison between the two studies is though, for methodological reasons, of limited value. Sarasjärvi and colleagues [21] found that a bifactor model had the best fit, with a strong general mental wellbeing factor and a weaker specific eudaimonic factor, but they also stated that they have discarded all factor models that are “inconsistent with any of the proposed latent structures of mental wellbeing”. This means that they only reported findings consistent with the assumed theory of which items that capture eudaimonic versus hedonic wellbeing. Since the group behind WEMWBS is not entirely clear on which items that are supposed to capture which aspect this is especially problematic. Item 3, 4, 5, and 7 constitute our factor 1. The word “relaxed” in item 3 is in the Swedish version translated into a word that normally is translated to the English word “calm” rather than “relaxed”. The Swedish word for “calm” describes an experience less physiological and more cognitive than the word “relaxed” does. This means factor 1 may be interpreted as a cognitive factor. There is, to our knowledge, no published information on the translation and adaptation procedure of the Swedish SWEMWBS and thus no documentation available regarding the linguistic equivalence of the Swedish version. Item 6 in the current study has a very low factor loading on both factors in the EFA. When it comes to the possible effect of culture on the factor structure of an instrument there is no particular reason why the factor structure should be different in Sweden in comparison to England, but “culture” is not only a question of geography but also a question of time; The material for this study was gathered during the time of the covid‐pandemic when people in general to a large extent were encouraged by the authorities to distance themselves socially due to the risk of getting ill. This might have affected the ratings, as item 6 asks whether one has felt close to other people. In the CFAs, the chi‐square statistic was significant for both models, which is expected given the large sample size and the sensitivity of *χ* ^2^ to sample size [22], and was therefore not interpreted as a primary indicator of model fit. The discrepancy between RMSEA and SRMR in the CFAs is also of interest, where the RMSEA was unacceptable in both models, whereas the SRMR was excellent in both models but better in the two‐factor model. This difference could reflect the data level, as previous research has suggested that SRMR might be a better option for ordinal data than RMSEA [23]. The results from the multi‐group CFAs indicate that SWEMWBS demonstrates metric measurement invariance across age groups for both the one‐factor and two‐factor models, suggesting that item loadings are comparable across age categories and that the scale functions similarly across the age range.

The most noticeable strength of the current study is the large, age‐stratified community‐based sample. According to the SWEMWBS homepage the instrument has been validated for people up to the age of 74. We know that the study by Melin and colleagues [9] had participants as old as 84, but the current study has now contributed with participants up to the age of 102 which is also a strength. There are, however, some limitations as well. The amount of questions comprising Liv och hälsa may have resulted in rater fatigue and influenced the answers. Since the questions of Kessler‐6 were placed right after the questions of SWEMWBS this has supposedly affected the measures quite equally. This might also be the case when it comes to the risk of those potential responders experiencing the lowest wellbeing and/or most psychological distress to a larger extent not answering the survey at all. The effect of the order of the measures is also unknown. The correlation of SWEMWBS to Kessler‐6 would have been more informative if the Swedish translation of Kessler‐6 was adequately validated. As mentioned above, Kessler‐6 has been investigated through comparison to the Swedish translation of GHQ‐12, but since GHQ‐12 is not well validated in Swedish either, this does not count as strong support for the validity of the Swedish translation of Kessler‐6. Another limitation was that the study did not include correlation to a measure meant to capture the same concept. This is because the study only included data from the survey at hand. To our knowledge there is also no such well validated instrument in Swedish capturing the same concept as SWEMWBS and that is part of why this study came about. The design of the study is cross‐sectional which means it is not possible to compare data from two different occasions. Considering that data was collected during an historically exception period of time (i.e., during the covid pandemic) this must be taken into consideration. For example the factor loadings of item 6 are, as mentioned, relatively low. Furthermore, although internal consistency reliability was examined, the cross‐sectional design made it impossible to assess test–retest reliability. Finally, the presence of systematic differences in missing data across age and educational level suggests that the assumption of completely random missingness is not fully met. This may have affected the representativeness of the final analytical sample, which should be considered when interpreting the generalizability of the findings to the broader population.

## 5 Conclusions

The official Swedish translation of SWEMWBS demonstrated good reliability and good convergent validity. Factor analyses indicated better statistical support for a two‐factor model compared to a one‐factor model, which contrasts with findings from the original English version but is in line with some previous studies on translated versions. However, the latent factors were very highly correlated, indicating substantial overlap between the dimensions and suggesting limited discriminant validity. This high degree of overlap, together with previous research supporting a unidimensional structure, suggests that the scale may best be interpreted as unidimensional in the Swedish context. The discrepancy may be due to language‐related differences in item interpretation or contextual factors related to data collection during the COVID‐19 pandemic. There is a need for further research on the Swedish translation of the WEMWBS, as existing validation studies remain inconclusive despite the instrument being widely used in large‐scale settings.

## Author Contributions

**Frida Ekbom Lodin:** conceptualization, writing – original draft, methodology, writing – review and editing, formal analysis. **Johnny Pellas:** conceptualization, writing – original draft, methodology, writing – review and editing, formal analysis, supervision.

## Funding

The authors have nothing to report.

## Conflicts of Interest

The authors declare no conflicts of interest.

## Transparency Statement

The lead author Johnny Pellas confirms that this manuscript is an honest, accurate, and transparent account of the study being reported; that no important aspects of the study have been omitted; and that any discrepancies from the study as planned have been explained.

## Data Availability

The data that support the findings of this study are available from Liv och hälsa. Restrictions apply to the availability of these data, which were used under license for this study. Data are available from the author(s) with the permission of Liv och hälsa.

## References

[1] Organization WH , “WHO Fact Sheets: Strengthening Mental Health Promotion,” (2001).

[2] The University of Warwick , “The Warwick‐Edinburgh Mental Wellbeing Scales ‐ WEMWBS,” (2025), https://warwick.ac.uk/services/innovations/wemwbs.

[3] R. M. Ryan and E. L. Deci , “On Happiness and Human Potentials: A Review of Research on Hedonic and Eudaimonic Well‐Being,” Annual Review of Psychology 52 (2001): 141–166. 10.1146/annurev.psych.52.1.141 11148302

[4] C. L. M. Keyes , “Mental Illness and/or Mental Health? Investigating Axioms of the Complete State Model of Health,” Journal of Consulting and Clinical Psychology 73, no. 3 (2005): 539–548. 15982151 10.1037/0022-006X.73.3.539

[5] J. B. Luoma , S. C. Hayes , and R. D. Walser , Learning ACT: An Acceptance & Commitment Therapy Skills Training Manual for Therapists, 2nd ed. (New Harbinger Publications, Incorporated, 2017).

[6] R. Tennant , L. Hiller , R. Fishwick , et al., “The Warwick‐Edinburgh Mental Well‐Being Scale (WEMWBS): Development and UK Validation,” Health and Quality of Life Outcomes 5 (2007): 63. 18042300 10.1186/1477-7525-5-63 PMC2222612

[7] S. Stewart‐Brown , A. Tennant , R. Tennant , S. Platt , J. Parkinson , and S. Weich , “Internal Construct Validity of the Warwick‐Edinburgh Mental Well‐Being Scale (WEMWBS): A Rasch Analysis Using Data From the Scottish Health Education Population Survey,” Health and Quality of Life Outcomes 7 (2009): 15. 19228398 10.1186/1477-7525-7-15 PMC2669062

[8] A. Haver , K. Akerjordet , P. Caputi , T. Furunes , and C. Magee , “Measuring Mental Well‐Being: A Validation of the Short Warwick‐Edinburgh Mental Well‐Being Scale in Norwegian and Swedish,” Scandinavian Journal of Public Health 43, no. 7 (2015): 721–727. 26041133 10.1177/1403494815588862

[9] J. Melin , A. Lundin , and M. Johansson , “An Off‐Target Scale Limits the Utility of Short Warwick‐Edinburgh Mental Well‐Being Scale (SWEMWBS) as a Measure of Well‐Being in Public Health Surveys,” Public Health 202 (2022): 43–48. 34883409 10.1016/j.puhe.2021.10.009

[10] L. E. Aarø , A. S. Fismen , B. Wold , et al., “Nordic Adolescents Responding to Demanding Survey Scales in Boring Contexts: Examining Straightlining,” Journal of Adolescence 94, no. 6 (2022): 829–843. 35719057 10.1002/jad.12066

[11] A. H. Pakpour , M. Eriksson , I. Erixon , et al., “The Short Warwick‐Edinburgh Mental Well‐Being Scale (SWEMWBS)—A Psychometric Evaluation of Adolescents in Sweden During the COVID‐19 Pandemic,” Heliyon 10, no. 6 (2024): e27620. 38510050 10.1016/j.heliyon.2024.e27620 PMC10950601

[12] Region Örebro län . Liv & hälsa (2024), https://utveckling.regionorebrolan.se/sv/valfard-och-folkhalsa/folkhalsan-i-siffror/liv--halsa/.

[13] R. C. Kessler , P. R. Barker , L. J. Colpe , et al., “Screening for Serious Mental Illness in the General Population,” Archives of General Psychiatry 60, no. 2 (2003): 184–189. 12578436 10.1001/archpsyc.60.2.184

[14] R. C. Kessler , J. G. Green , M. J. Gruber , et al., “Screening for Serious Mental Illness in the General Population with the K6 Screening Scale: Results From the WHO World Mental Health (WMH) Survey Initiative,” supplement, International Journal of Methods in Psychiatric Research 19, no. Suppl 1 (2010): 4–22. 20527002 10.1002/mpr.310 PMC3659799

[15] National comorbidity survey , “K10 and K6 Scales [date unknown]”, https://www.hcp.med.harvard.edu/ncs/k6_scales.php.

[16] A. Lundin , J. J. Muwonge , M. Lalouni , and J. Åhlén , “Measuring Psychological Distress Using the 12‐item General Health Questionnaire and the Six‐Item Kessler Psychological Distress Scale. Psychometric Comparison and Equipercentile Equating of the Two Scales,” International Journal of Methods in Psychiatric Research 33, no. 3 (2024): e2033. 38963772 10.1002/mpr.2033 PMC11223604

[17] M. T. Kalkbrenner , “Alpha, Omega, and H Internal Consistency Reliability Estimates: Reviewing These Options and When to Use Them,” Counseling Outcome Research and Evaluation 14, no. 1 (2023): 77–88.

[18] J. Cohen , “A Power Primer,” Psychological Bulletin 112, no. 1 (1992): 155–159. 19565683 10.1037//0033-2909.112.1.155

[19] L. E. van Zyl and P. M. Ten Klooster , “Exploratory Structural Equation Modeling: Practical Guidelines and Tutorial With a Convenient Online Tool for Mplus,” Frontiers in Psychiatry 12 (2022): 795672. 35069293 10.3389/fpsyt.2021.795672 PMC8779472

[20] F. F. Chen , “Sensitivity of Goodness of Fit Indexes to Lack of Measurement Invariance,” Structural Equation Modeling: A Multidisciplinary Journal 14, no. 3 (2007): 464–504.

[21] K. K. Sarasjärvi , M. Elovainio , K. Appelqvist‐Schmidlechner , P. Solin , N. Tamminen , and S. Therman , “Exploring the Structure and Psychometric Properties of the Warwick‐Edinburgh Mental Well‐Being Scale (WEMWBS) in a Representative Adult Population Sample,” Psychiatry Research 328 (2023): 115465. 37708805 10.1016/j.psychres.2023.115465

[22] L. Hu and P. M. Bentler , “Cutoff Criteria for Fit Indexes in Covariance Structure Analysis: Conventional Criteria Versus New Alternatives,” Structural Equation Modeling: A Multidisciplinary Journal 6, no. 1 (1999): 1–55.

[23] D. Shi , A. Maydeu‐Olivares , and Y. Rosseel , “Assessing Fit in Ordinal Factor Analysis Models: SRMR vs. RMSEA,” Structural Equation Modeling: A Multidisciplinary Journal 27, no. 1 (2020): 1–15.

